# Breast Cancer Screening Among Females With and Without Schizophrenia

**DOI:** 10.1001/jamanetworkopen.2023.45530

**Published:** 2023-11-29

**Authors:** Braden O’Neill, Abban Yusuf, Aisha Lofters, Anjie Huang, Ngozi Ekeleme, Tara Kiran, Michelle Greiver, Frank Sullivan, Paul Kurdyak

**Affiliations:** 1 MAP Centre for Urban Health Solutions, Li Ka Shing Knowledge Institute, St Michael’s Hospital, Unity Health Toronto, Ontario, Canada; 2 Department of Family and Community Medicine, St Michael’s Hospital, Toronto, Ontario, Canada; 3 Department of Family and Community Medicine, Temerty Faculty of Medicine, University of Toronto, Ontario, Canada; 4 Women’s College Research Institute, Toronto, Ontario, Canada; 5 ICES, Toronto, Ontario, Canada; 6 Department of Family and Community Medicine, North York General Hospital, Toronto, Ontario, Canada; 7 School of Medicine, Sir James Mackenzie Institute for Early Diagnosis, Population and Behavioural Science Division, University of St Andrews, St Andrews, Scotland; 8 Institute for Mental Health Policy Research and Campbell Family Mental Health Research Institute, Centre for Addiction and Mental Health, Toronto, Ontario, Canada

## Abstract

**Importance:**

Breast cancer screening with mammography is recommended in Ontario, Canada, for females 50 years or older. Females with schizophrenia are at higher risk of breast cancer, but in Ontario it is currently unknown whether breast cancer screening completion differs between those with vs without schizophrenia and whether primary care payment models are a factor.

**Objective:**

To compare breast cancer screening completion within 2 years after the 50th birthday among females with and without schizophrenia, and to identify the association between breast cancer screening completion and different primary care payment models.

**Design, Setting, and Participants:**

This case-control study analyzed Ontario-wide administrative data on females with and without schizophrenia who turned 50 years of age between January 1, 2010, and December 31, 2019. Those with schizophrenia (cases) were matched 1:10 to those without schizophrenia (controls) on local health integration network, income quintile, rural residence, birth dates, and weighted Aggregated Diagnosis Group score. Data analysis was performed from November 2021 to February 2023.

**Exposures:**

Exposures were schizophrenia and primary care payment models.

**Main Outcomes and Measures:**

Outcomes included breast cancer screening completion among cases and controls within 2 years after their 50th birthday and the association with receipt of care from primary care physicians enrolled in different primary care payment models, which were analyzed using logistic regression and reported as odds ratios (ORs) and 95% CIs.

**Results:**

The study included 11 631 females with schizophrenia who turned 50 years of age during the study period and a matched cohort of 115 959 females without schizophrenia, for a total of 127 590 patients. Overall, 69.3% of cases and 77.1% of controls had a mammogram within 2 years after their 50th birthday. Cases had lower odds of breast cancer screening completion within 2 years after their 50th birthday (OR, 0.67; 95% CI, 0.64-0.70). Cases who received care from a primary care physician in a fee-for-service (OR, 0.57; 95% CI, 0.53-0.60) or enhanced fee-for-service (OR, 0.79; 95% CI, 0.75-0.82) payment model had lower odds of having a mammogram than cases whose physicians were paid under a Family Health Team model.

**Conclusions and Relevance:**

This case-control study found that, in Ontario, Canada, breast cancer screening completion was lower among females with schizophrenia, and differences from those without schizophrenia may partially be explained by differences in primary care payment models. Widening the availability of team-based primary care for females with schizophrenia may play a role in increased breast cancer screening rates.

## Key Points

**Question:**

How does breast cancer screening completion in Ontario, Canada, differ between females with and without schizophrenia, and how does it compare among those who access care from clinicians who work under different primary care payment models?

**Findings:**

In this case-control study of 127 590 females with schizophrenia (cases) and without (matched controls) schizophrenia, fewer cases had a mammogram within 2 years of their 50th birthday compared with controls. A higher proportion of cases whose clinicians were enrolled in a blended capitation payment model completed mammograms compared with cases whose clinicians were enrolled in fee-for-service or enhanced fee-for-service payment models.

**Meaning:**

Findings of this study suggest that females with schizophrenia tend to undergo less breast cancer screening compared with females without schizophrenia; some of these differences are associated with differences in primary care payment models.

## Teaser

This case-control study examines the association between schizophrenia diagnosis, mammography rate, and physician reimbursement structures in Ontario, Canada.

## Introduction

People with schizophrenia experience markedly earlier mortality than the general population, dying 10 to 25 years sooner than those without the condition.^1,2^ Multiple studies of premature mortality among this population have identified cancer as an important factor.^3,4^ Schizophrenia is 1 of the top 5 mental health conditions with the largest implications for the health of people in Ontario, Canada.^5^

People with schizophrenia may be at higher risk of developing breast cancer. A study in Finland reported that females with schizophrenia had higher rates of breast cancer, especially those with antipsychotic medication use for at least 5 years.^6^ A meta-analysis of 125 760 patients showed that those with schizophrenia had a 31% increased risk of developing breast cancer (standardized incidence ratio, 1.31; 95% CI, 1.14-1.50).^7^ The association between schizophrenia and breast cancer may be partially attributable to a shared genetic cause between the 2 diseases.^8^

Cancer screening, including for cervical and colorectal cancers, is a factor in reduced mortality.^9,10^ Although uncertainty exists about the effectiveness of mammography to reduce breast cancer–specific or all-cause mortality,^11,12,13^ it is recommended by Cancer Care Ontario and the Canadian Task Force on Preventive Health Care.^14,15^ In Ontario, the standard of care and guideline recommendation for patients with an average risk of breast cancer is screening with mammography every 2 years from age 50 to 74 years.^16^

Many jurisdictions, including Ontario, have health system–level cancer screening programs, which are known to have differential access by socioeconomic status.^17^ Some studies have shown lower cancer screening rates among people with severe mental illness, including 2 Ontario studies: 1 reporting lower cervical cancer screening rates among people with psychosis in a Toronto Family Health Team,^18^ and another reporting lower cervical cancer screening among people with schizophrenia from provincewide data.^19^ An international systematic review found that females with schizophrenia across multiple countries were half as likely to be screened for breast cancer than the general population, but the study did not include subgroup or sensitivity analyses of the characteristics of the study settings, such as different features of how health systems were organized or funded that may be associated with screening completion.^20^ Another systematic review found that people with psychosis had a higher risk of breast cancer and were 22% more likely to have had metastasized cancer at the time of diagnosis.^21^ Studies from the US^22,23,24^ and the UK^25,26^ and 2 systematic reviews^27,28^ found lower cancer screening among people with serious mental illness. Studies from Manitoba, Canada, identified lower breast cancer screening with mammography^29^ and lower cervical cancer screening with Papanicolaou tests among people with schizophrenia.^30^ Although these studies reported differences in cancer screening rates between people with and without schizophrenia, they did not focus on aspects of health system delivery, such as primary care payment models or care organization, that could play a role in increased cancer screening among this population.

There are differences between the health systems in previous studies and the Ontario setting that highlight the importance of investigating screening rates among people with schizophrenia in the Ontario setting. Starting in 2002, Ontario family physicians (who provide most primary care in the province) have had the option to enter a series of new primary care payment models. These models included enhanced fee-for-service (FFS), known as Family Health Groups (FHGs) and comprehensive care models, whereby physicians receive pay-for-performance financial incentives for preventive care, such as completion of cancer screening. Another available model, known as Family Health Organization (FHO), provides compensation mostly through blended capitation rather than FFS payments in addition to pay-for-performance preventive care incentives.^31^ In a FHO, specific pay-for-performance financial incentives were instituted starting in 2006 for preventive care, such as cervical, breast, and colon cancer screening. Some FHOs are part of Family Health Teams (FHTs), with additional team members such as nurses, social workers, dietitians, and other allied health professionals. In 2016, 29.1% of Ontario family physicians were in an FFS model, 23.8% were in enhanced FFS models, and 23.7% were in FHO-FHT models.^32^ Research comparing cancer screening rates between patients who accessed care from physicians in these capitation-based models and those in the traditional FFS model did not find a difference in rates.^33^ Additionally, there were no substantial differences between these models in quality of care for other conditions among the general population, such as those with diabetes,^34^ and 1 study^35^ suggested that timely access to care might be worse for people whose clinicians were under the capitated models. However, among those with schizophrenia, there is evidence of better guideline-congruent diabetes care favoring capitated models.^36^ Therefore, it is important to understand the extent to which these capitation and team-based payment initiatives may be beneficial for cancer screening among high-risk populations, such as those with schizophrenia.

The present study aimed to compare breast cancer screening (mammogram) completion within 2 years after the 50th birthday among females with and without schizophrenia and to identify the association between breast cancer screening completion and different primary care payment models in Ontario, Canada. We investigated differences in breast cancer screening completion among those with schizophrenia who accessed care from a physician practicing in a capitated model vs an FFS model, and differences in rates between capitated models. We hypothesized that a capitated model would have patients with higher breast cancer screening completion, whereas a team-based capitated model would have patients with the highest breast cancer screening completion.

## Methods

This retrospective matched case-control study obtained data from ICES, which securely houses and provides facility for analyzing health administrative data from Ontario, including data cleaning and linkage. ICES is a prescribed entity under the Section 45 provision in the Ontario Personal Health Information Protection Act, which authorizes health information custodians to transfer personal health information for evaluation of health services for resource allocation planning. In accordance with the Section 45 provision, this study was exempt from research ethics board approval and informed consent requirement. We followed the Strengthening the Reporting of Observational Studies in Epidemiology (STROBE) and Reporting of Studies Conducted Using Observational Routinely Collected Data (RECORD) reporting guidelines.

### Setting

Ontario is Canada’s most populous province, with a population of 15 007 816 as of 2022 (approximately 40% of Canada’s population).^37^ All necessary physician visits, medical tests, hospital services, and cancer screenings (including mammograms) are fully insured by the Ontario Health Insurance Plan (OHIP) for all Ontario permanent residents, with no payment at the point of care. Primary care physician (PCP) services are paid for by OHIP through several primary care payment models; reform of these models was instituted between 2002 and 2007.^38^

As described, the 3 primary care payment models in Ontario are as follows: FFS, in which PCPs receive payment per visit, without pay-for-performance incentives; enhanced FFS (FHG), in which most compensation is from payment per visit, with some pay-for-performance incentives; and capitation (FHO-FHT), in which most compensation is from a per-patient per-year payment, with pay-for-performance incentives for preventive care.^39^ The FHT model includes additional per-patient funding for hiring nonphysician staff, such as nurses, dietitians, and social workers.

### Study Design and Patient Population

The study population included all Ontario residents who were documented as female in the Registered Persons Database, who had continuous OHIP coverage throughout the study period (January 1, 2010, to December 31, 2019), and who turned 50 years of age during the study period. The primary analysis compared breast cancer screening completion between females with schizophrenia and those without that condition. To identify schizophrenia status, we used the algorithm developed by Kurdyak et al,^40^ including data from outpatient physician visits and hospitalizations to identify documentation at multiple time points and settings of a schizophrenia diagnosis.

We excluded patients who were diagnosed with breast cancer before age 50 years, as identified through relevant OHIP codes in the Ontario Cancer Registry, a structured database in which all cancer diagnoses in Ontario are documented. Additionally, we excluded those who had mastectomy prior to age 50 years and received breast implants. We excluded females with particularly high risk for breast cancer, as identified from breast cancer screening that was organized through the High Risk Ontario Breast Screening Program for females with a known personal or first-degree family history of a gene variant associated with breast cancer, who were previously assessed by a genetics clinic as having a greater than 25% lifetime risk, those with a personal or family history of a cancer suggestive of a hereditary breast cancer syndrome, and those with a personal history of chest radiation before age 30 years.^41^

### Outcomes and Covariates

The primary outcome was completion of breast cancer screening within 2 years after the 50th birthday. We identified this status from the Ontario Breast Screening Program,^16^ which facilitates breast cancer screening completion for females aged 50 to 74 years with average risk (excluding those with a history of breast cancer, with a high risk of breast cancer, or with breast implants). We also identified completion of breast cancer screening from physician billing codes in the OHIP database indicating that a radiologist had read and reported the results of a screening mammogram; there are different codes for diagnostic mammograms.

Cases (females with schizophrenia) and controls (females without schizophrenia) were matched 1:10 on the following variables: local health integration network (the region in which the person lives in the province, as of January 1, 2010),^42^ income quintile (1-5, with 1 indicating the lowest income and 5 indicating the highest income), rural residence (residential address in a community with <10 000 people as of January 1, 2010), birth dates within 180 days of each other, and weighted Aggregated Diagnosis Group (ADG) score.^43^

Data about age and rurality were obtained from the Registered Persons Database. Income levels were ascertained by using Canadian Census data and by assigning residential-address forward sortation areas to income quintiles using the Statistics Canada Postal Code Conversion File Plus.^44,45^ Health status was assessed using ADGs (Johns Hopkins ACG System).^43^ These ADGs allocated related diseases and reasons for presentation to health care to individual ADGs according to the following characteristics: duration, severity, diagnostic certainty, cause, and specialty care involvement. Data used to calculate ADGs were generated when patients interacted with any part of the health system, including primary, specialty outpatient, and hospital and community care. These groupings were associated with different levels of future health service use and represented a measure of patient complexity. Health service use was assessed from OHIP physician billing codes related to the type of service, and data were obtained from the Discharge Abstract Database,^46^ National Ambulatory Care Reporting System,^47^ and the Ontario Mental Health Reporting System.^48^

Data on primary care payment models were obtained from the Client Agency Program Enrolment data set.^49^ Cases and controls were attributed to a physician if they were formally enrolled (rostered) or, for those receiving care from physicians who were not under capitation models, were assigned to the family physician who billed the largest dollar amount for primary care services for that patient during the study period.^50^ We considered the following primary care payment models in this study: team-based capitation (FHT), non–team-based capitation (FHO), enhanced FFS/FHG, physician not in a patient enrollment model (FFS physicians), and no physician (patient did not have any primary care visits during the study period and were not designated as rostered to a PCP in a capitated payment model). More information on the variables extracted from each database is provided in eTables 1 and 2 in Supplement 1.

### Statistical Analysis

The accuracy of matching cases to controls was assessed using weighted SD of differences between groups. Baseline characteristics (such as income quintiles) were reported with descriptive statistics for both cases and controls. The outcome of breast cancer screening completion for cases and controls was analyzed using logistic regression and reported with odds ratios (ORs). Furthermore, using logistic regression and reported with ORs, we conducted an unadjusted analysis to compare breast cancer screening completion among people with schizophrenia across primary care payment models.

Significance testing was performed with 2-sided tests. *P* < .05 was used to indicate statistical significance. Data analysis was performed from November 2021 to February 2023 using SAS, version 9.4 (SAS Institute Inc).

## Results

**Figure 1.**
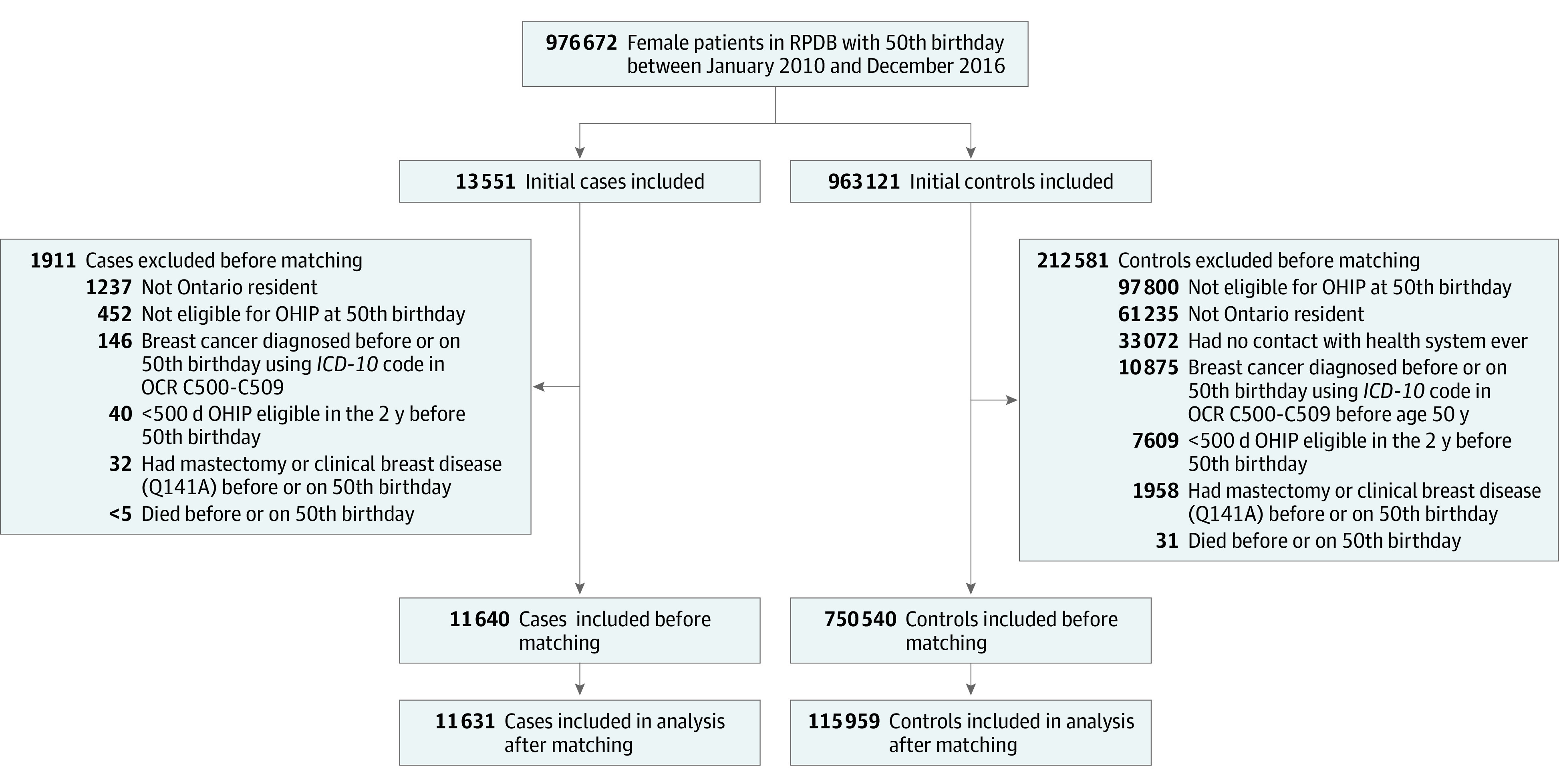
Participant Flowchart *ICD-10* indicates *International Statistical Classification of Diseases and Related Health Problems, Tenth Revision*; OCR, Ontario Cancer Registry; OHIP, Ontario Health Insurance Plan; RPDB, Registered Persons Database.

This study included 11 631 females with schizophrenia (cases) who turned 50 years of age during the study period and were matched to 115 959 without schizophrenia (controls), for a total of 127 590 participants (Figure 1). Matching was adequate, with SDs close to 0 (Table 1). Overall, 34.8% of cases and 34.9% of controls were in the lowest income quintile, and 8.7% of cases and 8.6% of controls lived in rural communities. The largest proportion of both cases (13.1%) and controls (8.6%) lived in the Toronto Central region, and 1.9% of cases and controls lived in the rural Northwest region of Ontario. Most females with schizophrenia (46.2%) had a weighted ADG score of 10 or higher, suggesting substantial comorbidity and future health service use.

**Table 1.** Sociodemographic and Clinical Characteristics of Females With Schizophrenia vs Matched People Without Schizophrenia

Characteristic	No. (%)			*P* value^a^	Weighted SD
	Females with schizophrenia (n = 11 631)	Females without schizophrenia (n = 115 959)	Total		
Completed mammogram within 2 y after 50th birthday	8055 (69.3)	89 405 (77.1)	97 460 (76.4)	<.001	0.18
Completed mammogram before 50th birthday	6468 (55.6)	73 328 (63.2)	79 796 (62.5)	<.001	0.16
Income quintile^b^					
Missing data	52 (0.4)	306 (0.3)	358 (0.3)	NA	0
1	4048 (34.8)	40 441 (34.9)	44 489 (34.9)	NA	0
2	2467 (21.2)	24 662 (21.3)	27 129 (21.3)	NA	0
3	1998 (17.2)	19 952 (17.2)	21 950 (17.2)	NA	0
4	1650 (14.2)	16 442 (14.2)	18 092 (14.2)	NA	0
5	1416 (12.2)	14 156 (12.2)	15 572 (12.2)	NA	0
Rural residence					
Missing data	19 (0.2)	91 (0.1)	110 (0.1)	NA	0
Yes	1008 (8.7)	9951 (8.6)	10 959 (8.6)	NA	0
No	10 604 (91.2)	105 917 (91.3)	116 521 (91.3)	NA	0
Weighted ADG score without ADG 24 or ADG 25^c^					
Mean (SD)	10.8 (10.7)	9.9 (9.7)	10.0 (9.8)	NA	0.09
Median (IQR)	8 (3-18)	8 (2-17)	8 (2-17)	NA	0.09
≤5	4369 (37.6)	43 627 (37.6)	47 996 (37.6)	NA	0
6-9	1885 (16.2)	18 777 (16.2)	20 662 (16.2)	NA	0
≥10	5377 (46.2)	53 555 (46.2)	58 932 (46.2)	NA	0
Primary care payment models					
Unattributable to any payment model	733 (6.3)	8598 (7.4)	9331 (9.5)	NA	NA
No physician	688 (5.9)	4814 (4.2)	5502 (4.3)	<.001	0.08
Non-FHT FHO	2874 (24.7)	30 938 (26.7)	33 812 (26.5)	<.001	0.05
Enhanced FFS/FHG	3583 (30.8)	36 269 (31.3)	39 852 (31.2)	<.001	0.01
FHT	2880 (24.8)	27 741 (23.9)	30 621 (24.0)		0.02
FFS	873 (7.5)	7599 (6.6)	8472 (6.6)	<.001	0.04

Abbreviations: ADG, Aggregated Diagnosis Group; FFS, fee-for-service; FHG, Family Health Group; FHO, Family Health Organization; FHT, Family Health Team; NA, not applicable.

^a^ *P* values were calculated using χ^2^ tests.

^b^ Quintile 1 indicated lowest income and 5 indicated highest income.

^c^ The ADG 24 score indicated psychosocial, recurrent or persistent, stable. The ADG 25 score indicated psychosocial, recurrent or persistent, unstable. Both scores are associated with diagnosis of schizophrenia.

For the primary outcome of breast cancer screening completion, 69.3% of cases and 77.1% of controls had a mammogram within 2 years of their 50th birthday. Those with schizophrenia had lower odds of having a mammogram compared with those with schizophrenia (OR, 0.67; 95% CI, 0.64-0.70; *P* < .001) (Table 2).

**Table 2.** Comparison of Breast Cancer Screening Completion Before and After 50th Birthday Between Females With vs Without Schizophrenia

Outcome	OR (95% CI)^a^	*P* value
Breast cancer screening completion within 2 y after 50th birthday, with vs without schizophrenia	0.67 (0.64-0.70)	<.001
Breast cancer screening completion before 50th birthday, with vs without schizophrenia	0.73 (0.70-0.76)	<.001

Abbreviation: OR, odds ratio.

^a^ The ORs were calculated using logistic regression.

**Figure 2.**
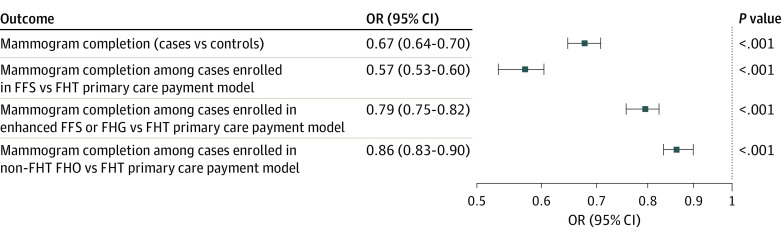
Breast Cancer Screening Completion Within 2 Years After 50th Birthday Between Cases and Controls and Among Cases by Primary Care Payment Models FFS indicates fee-for-service; FHG, Family Health Group; FHO, Family Health Organization; FHT, Family Health Team; OR, odds ratio.

There were differences in breast cancer screening completion among cases who received care from PCPs in different primary care payment models (Table 3; Figure 2). Most cases were enrolled with a physician either in an FHG model (30.8%) or an FHT model (24.8%) (Table 1). Among females with schizophrenia, 5.9% were found to have no physician visits during the study period. These patients also had lower odds of having a mammogram while being enrolled with a physician in an FFS vs an FHT model (OR, 0.57; 95% CI, 0.53-0.60; *P* < .001). The odds of having a mammogram while enrolled with a physician in an FHG model were lower compared with an FHT model for females with schizophrenia (OR, 0.79; 95% CI, 0.75-0.82; *P* < .001).

**Table 3.** Comparison of Breast Cancer Screening Among Females With Schizophrenia Enrolled in Different Primary Care Payment Models

Primary care payment model	OR (95% CI)^a^	*P* value
FFS vs FHT	0.57 (0.53-0.60)	<.001
Enhanced FFS/FHG vs FHT	0.79 (0.75-0.82)	<.001
Non-FHT FHO vs FHT	0.86 (0.83-0.90)	<.001

Abbreviations: FFS, fee-for-service; FHG, Family Health Group; FHO, Family Health Organization; FHT, Family Health Team; OR, odds ratio.

^a^ The ORs were calculated using logistic regression.

Most of the total study population (62.5%) had a mammogram before age 50 years. Furthermore, 55.6% of cases had a mammogram before age 50 years. The proportion of controls who had a mammogram before age 50 years was higher than the proportion of cases (63.2%).

## Discussion

This case-control study of breast cancer screening among females in Ontario, Canada, found lower odds of undergoing mammograms among those with schizophrenia. The overall pattern of lower completion of breast cancer screening among patients with schizophrenia was consistent with findings in other settings.

We explored differences in breast cancer screening completion between patients who accessed care from physicians in different primary care payment models to identify associations between these models and breast cancer screening completion. We found higher odds of mammogram completion among those receiving care under capitated models, in which most of the payments were per patient per year (rather than per visit) and there were pay-for-performance incentives for high proportions of breast cancer screening completion. We were unable to assess the relative implications of these 2 aspects of compensation for breast cancer screening completion, but the fact that patients of physicians in capitation models had higher odds of having mammograms suggests an association with 1 or both aspects of of these models. This association with breast cancer screening was not seen in the general Ontario population in the year after the pay-for-performance initiative was instituted^51^ (ie, 63.2% of eligible patients had a mammogram within 30 months of March 31, 2010). In the present study, we found a higher proportion of breast cancer screening completion (77.1% of those without schizophrenia and 69.3% of those with schizophrenia). One possible explanation for this higher mammogram completion may be the use of different definitions or may be the improvement, over time, in breast cancer screening completion within capitation models, specifically patients with higher barriers to screening completion, such as those with schizophrenia. We believe the higher breast cancer screening completion in this study among females with schizophrenia receiving care from PCPs in capitation and team-based capitation models compared with 2010 data may be associated with different allocation of resources (eg, physician or allied health professional time); this resource allocation may be particularly beneficial for patients with complex care needs, such as those with schizophrenia. A recent study in Ontario comparing primary care enrollment of adults with and without serious mental illness found lower enrollment in those models among people with serious mental illness.^52^ The finding that a mammogram was higher among those with schizophrenia in capitation models (which require enrollment) suggests that ensuring people with schizophrenia have access to these models is warranted. Total health care costs have been shown to be lower among patients of physicians in capitation models than FFS models, further supporting this point,^53^ although a specific comparison between costs for people with schizophrenia between those models has not been reported.

One finding, which to our knowledge has not been reported previously, was the proportion of people in both the case and control cohorts who had mammograms before the age of 50 years. Ontario guidelines recommend the completion of breast cancer screening for people with average risk after age 50 years, noting that before age 50 years mammograms can be ordered for screening purposes on a case-by-case basis and in consultation between patients and clinicians. We found that 55.6% of cases and 63.2% of controls received mammograms before age 50 years. This finding represents a deviation from the Ontario guidelines and is likely associated with patient preference or concern about a family history of breast cancer leading to mammogram ordering at a younger age than at the age when routine screening is recommended. Since mammography has a lower positive predictive value for cancer detection among younger people given their lower prevalence of breast cancer,^54^ it is important that clinicians discuss the benefits and risks of this approach with patients.

### Limitations

Limitations of this study include the nature of observational data, which prevented our assessment of causality. We included only patients with valid Ontario health coverage and who were permanent residents of Ontario. Some variables were neighborhood level rather than individual level, such as income quintile, and thus we were unable to account for some potential confounders, such as race and ethnicity. Our definition of completing a screening mammogram was different from that used in other studies. We chose within 2 years after the 50th birthday because that was consistent with Ontario guidelines of starting breast cancer screening with mammography at age 50 years.

## Conclusions

This case-control study found that females with schizophrenia had lower breast cancer screening completion in Ontario, Canada, than those without schizophrenia. Among the cases, higher odds of mammography completion were seen in those who accessed care from PCPs who were paid under capitation rather than FFS; mammogram completion was highest among those who received care from PCPs working under team-based capitation models. Given that cancer mortality is one of the most substantial factors of mortality in people with schizophrenia, efforts to increase breast cancer screening rates are essential. Widening the availability of team-based, capitated primary care payment model may be a way to achieve this goal.

## Notes

Supplement 1. **eTable 1.** Databases Used in Study **eTable 2.** Definitions of Primary Care Models

Supplement 2.
Data Sharing Statement
