# Supplementary material for: Breast Cancer Screening Among Females With and Without Schizophrenia
Source: JAMA Netw Open. 2023 Nov 29;6(11):e2345530. doi: 10.1001/jamanetworkopen.2023.45530 (PMC10687664; doi:10.1001/jamanetworkopen.2023.45530)
Supplement: Supplement 1. — eTable 1. Databases Used in Study eTable 2. Definitions of Primary Care Models [file jamanetwopen-e2345530-s001.pdf]

Supplemental Online Content

O'Neill B, Yusuf A, Lofters A, et al. Breast cancer screening among females with and without schizophrenia. *JAMA Netw Open*. 2023;6(11):e2345530. doi:10.1001/jamanetworkopen.2023.45530

eTable 1. Databases Used in Study

eTable 2. Definitions of Primary Care Models

This supplemental material has been provided by the authors to give readers additional information about their work.

© 2023 O'Neill B et al. *JAMA Network Open*

eTable 1. Databases Used in Study

Database	Description	Time period searched	Outcomes from this database	Covariates from this database
OBSP	Ontario Breast Screening Program	January 2010-December 2019	Mammography	
DAD	Health Services Database	January 2010-December 2019		Aggregated Diagnosis Groups (ADG) calculated using Johns Hopkins Adjusted Clinical Groups (ACG) system; number of emergency department visits for psychiatric conditions, number of emergency department visits for non-psychiatric conditions
OHIP	Ontario Health Insurance Plan claims	January 2010-December 2019		Aggregated Diagnosis Groups (ADG) calculated using Johns Hopkins Adjusted Clinical Groups (ACG) system; number of primary care visits; number of primary mental health care visits; number of primary non-menta health care visits; number of outpatient psychiatric visits; number of psychiatric hospitalizations; physician billing codes indicating

				that radiologist read and reported a screening mammogram
SDS	Same Day Surgery Database	January 2010-December 2019		Health Services
OMHRS	Ontario Mental Health Reporting System	January 2010-December 2019		Number of psychiatric hospitalizations
NACRS	National Ambulatory Care Reporting System	January 2010-December 2019		Number of emergency department visits for psychiatric conditions, number of emergency department visits for non-psychiatric conditions
CPDB	Corporate Provider Database	January 2010-December 2019		Care Providers (Physician contract model)
CENSUS	Ontario Census Area Profiles	January 2010-December 2019		Income levels data
RPBD	Registered Persons Database	January 2010-December 2019		Age and rurality data
LHIN	Local Health Integration Network	January 2010-December 2019		Geography data
PCCF	Postal Code Conversion File	January 2010-December 2019		Postal codes to organise income quintiles and ethnicity quintiles
CAPE	Client Agency Program Enrolment	January 2010-December 2019		Used to identify where cases and controls are rostered in team-based primary care models
OCR	Ontario Cancer Registry	January 2010-December 2019		Used to exclude patients with breast cancer who would not have been eligible for screening mammogram

© 2023 O'Neill B et al. *JAMA Network Open*

PCPOP	Primary Care Population	2010-2016		Physician roster sizes and identification of primary care physicians

© 2023 O'Neill B et al. *JAMA Network Open*

eTable 2. Definitions of Primary Care Models

Primary care model	Definition
Family Health Team (FHT)	FHTs are organizations that are comprised of family physicians, nurse practitioners, registered nurses, social workers, etc. who collaborate to meet primary health care for their community (1)
Non-FHT FHO (Family Health Organization)	FHO models have 3 or more physicians enrolled in an organization, and receive payment chiefly from capitation, and otherwise through FFS payments (2)
FFS (Fee-for-Service)	In FFS, physician payment is based on volume of individual services provided (2)
Enhanced FFS/FHG (Enhanced Fee-for-Service/Family Health Group)	Physicians compensated primarily through FFS, but also eligible for bonuses and/or premiums based on patient enrollment (2)

Definition of primary exposure (diagnosis of schizophrenia)

Diagnosis of schizophrenia either from hospitalization:

From CIHI-Discharge Abstract Database
Incidence of: ICD-9 codes: 295, 298
Or ICD-10 codes: F20, F25, F29

AND/OR
From Ontario Mental Health Recording System (OMHRS)
Incidence of ICD-9 codes: 295, 298

OR if 3 physician visits for schizophrenia within a 36-month period

© 2023 O'Neill B et al. *JAMA Network Open*

Kurdyak et al.’s algorithm (3) was used to identify people with schizophrenia.

Definition of Primary outcome

Incident breast cancer screening as defined in OHIP database by:
Physician billing code of X185, X172, X178 (denoting screening mammography completion) or a Q-code of Q131A (‘family medicine’ billing code denoting completion of screening mammography), or had been screened through the Ontario Breast Screening Program (OBSP)

© 2023 O’Neill B et al. *JAMA Network Open*

eReferences

1. Government of Ontario: Ministry of Health and Long-Term Care. Family Health Teams [Internet]. [cited 2023 May 8]. Available from: https://www.health.gov.on.ca/en/pro/programs/fht/
2. Government of Ontario, Ministry of Health and Long-Term Care. Primary Care Payment Models in Ontario. Government of Ontario, Ministry of Health and Long-Term Care; 2020.
3. Kurdyak P, Lin E, Green D, Vigod S. Validation of a Population-Based Algorithm to Detect Chronic Psychotic Illness. Can J Psychiatry. 2015 Aug;60(8):362–8.

© 2023 O'Neill B et al. *JAMA Network Open*
