# Supplementary material for: Breast Cancer Screening Among Females With and Without Schizophrenia
Source: JAMA Netw Open. 2023 Nov 29;6(11):e2345530. doi: 10.1001/jamanetworkopen.2023.45530 (PMC10687664; doi:10.1001/jamanetworkopen.2023.45530)
Supplement: Supplement 2. — Data Sharing Statement [file jamanetwopen-e2345530-s002.pdf]

Data Sharing Statement

O'Neill. Breast Cancer Screening Among Females With and Without Schizophrenia. *JAMA Netw Open*. Published November 29, 2023. doi:10.1001/jamanetworkopen.2023.45530

Data
Data available: No

Additional Information
Explanation for why data not available: Data used in this study are from ICES, which consist of record-level, coded and linkable health data sets from the population of Ontario. These data are held securely and are not publicly available.
